# Impact of Health Workers’ Choice of COVID-19 Vaccine Booster on Immunization Levels in Istanbul, Turkey

**DOI:** 10.3390/vaccines11050935

**Published:** 2023-05-03

**Authors:** Meryem Merve Ören, Sevgi Canbaz, Sevim Meşe, Ali Ağaçfidan, Ömer Serdil Demir, Esra Karaca, Ayşe Rumeysa Doğruyol, Gökçe Hazar Otçu, Tufan Tükek, Nuray Özgülnar

**Affiliations:** 1 Department of Public Health, Istanbul Faculty of Medicine, Istanbul University, Istanbul 34116, Turkey; meryem.oren@istanbul.edu.tr (M.M.Ö.);; 2 Department of Medical Microbiology, Istanbul Faculty of Medicine, Istanbul University, Istanbul 34116, Turkey; 3 Department of Internal Medicine, Istanbul Faculty of Medicine, Istanbul University, Istanbul 34116, Turkey; tufan.tukek@istanbul.edu.tr

**Keywords:** BNT162b2 vaccine, CoronaVac vaccine, booster vaccination, healthcare workers, antibody responses

## Abstract

Background: There are limited data regarding short- and medium-term IgG antibody levels after the CoronaVac and BNT162b2 vaccines. This study aimed to investigate the antibody responses of health workers who initially received two doses of CoronaVac one month apart followed by a booster dose of either CoronaVac or BNT162b2, as well as determine whether either vaccine provided superior results. Methods: This research represents the second phase of a mixed-methods vaccine cohort study and was conducted between July 2021 and February 2022. The participants (n = 117) were interviewed in person and blood samples were collected before and at 1 and 6 months after the booster vaccination. Results: BNT162b2 was found to have greater immunogenic potential than CoronaVac (*p* < 0.001). Health workers without chronic disease exhibited statistically significant increases in antibody levels after both vaccines (*p* < 0.001), whereas only BNT162b2 caused a significant increase in antibody levels in participants with chronic disease (*p* < 0.001). Samples obtained before and at 1 and 6 months after the booster vaccination revealed no age- or sex-based differences in IgG-inducing potential for either vaccine (*p* > 0.05). Antibody levels were comparable in both vaccine groups before the booster regardless of COVID-19 history (*p* > 0.05); however, antibody levels were significantly higher after the BNT162b2 booster at 1 month (<0.001) and at 6 months, except among participants who had a positive history of COVID-19 infection (*p* < 0.001). Conclusions: Our results suggest that even a single booster dose of BNT162b2 after initial vaccination with CoronaVac provides a protective advantage against COVID-19, especially for risk groups such as health workers and those with chronic diseases.

## 1. Introduction

COVID-19, the disease caused by severe acute respiratory syndrome coronavirus 2 (SARS-CoV-2), was declared a pandemic by the World Health Organization (WHO) on 11 March 2020. On the same day, the Turkish Ministry of Health reported the first case of COVID-19 in Turkey, and the first death in Turkey occurred on 15 March 2020. As of 30 November 2022, more than 17 million COVID-19 cases and 100 thousand deaths had been reported in Turkey since the start of the pandemic [1].

As the most serious global health crisis of the last century, the COVID-19 pandemic required dramatic preventive measures worldwide. In the early stages, disease control efforts focused on the principles of lockdown/quarantine and the use of masks, hygiene, and social distancing. Later in the pandemic, vaccination programs were launched with vaccines that were granted emergency approval [2]. In Turkey, a national vaccination strategy was determined, and the administration of the first coronavirus vaccine (CoronaVac) began on 14 January 2021. Priority groups were vaccinated first based on assessments of risk and the adverse effects of the disease on the functioning of society [3].

As with other infectious pandemics, COVID-19 placed a great burden on national health systems. Adequate healthy staff are necessary to provide and maintain continuity of quality healthcare, in addition to protecting and promoting public health [4,5]. Health workers were at high risk of COVID-19 exposure during the diagnosis and treatment of patients with COVID-19, and the Occupational Health and Safety Administration (OSHA) classified health workers in the very-high- and high-risk groups during the COVID-19 pandemic [5]. An early report from Italy published on 19 June 2020 stated that 29,174 health workers had contracted the disease, accounting for 12% of all cases [6]. Therefore, health workers were among the first group to be vaccinated in Turkey. Within the first week after vaccination began in Turkey, 830,000 health workers received a first dose of the CoronaVac vaccine, followed by a second dose of CoronaVac one month later [3]. On 12 April 2021, the messenger RNA (mRNA) vaccine BNT162b2 was also introduced in our country. After that date, the public (including health workers) were able to choose which vaccine they received [2].

Although various vaccination strategies have been implemented in different countries, vaccination is known to be the most effective method for preventing infectious diseases. There are some studies in the literature demonstrating the duration of short-term immunity after COVID-19 vaccination [7,8], but the persistence of immunity in the long term remains uncertain [9,10]. Studies conducted on healthcare workers who became immune after infection have shown a decline in antibody levels over a 6-month period [11]; however, the degree and duration of immunity after vaccination against COVID-19 compared to natural immunity also remain unclear [7].

This study aimed to compare levels of antibody responses in health workers who first received 2 doses of the CoronaVac vaccine at an interval of 1 month, followed by a booster dose of CoronaVac or BNT162b2, and to identify any superiority between the vaccines.

## 2. Materials and Methods

### 2.1. Study Design and Participants

This study was conducted on a vaccine cohort that received two doses of the CoronaVac COVID-19 vaccine. The prospective, mixed-methods research included individuals who volunteered to participate in the study (nested group) from the vaccine cohort of 2493 individuals, and comprises both quantitative and qualitative dimensions. The qualitative aspect of the research is presented in another article.

**Figure 1:**
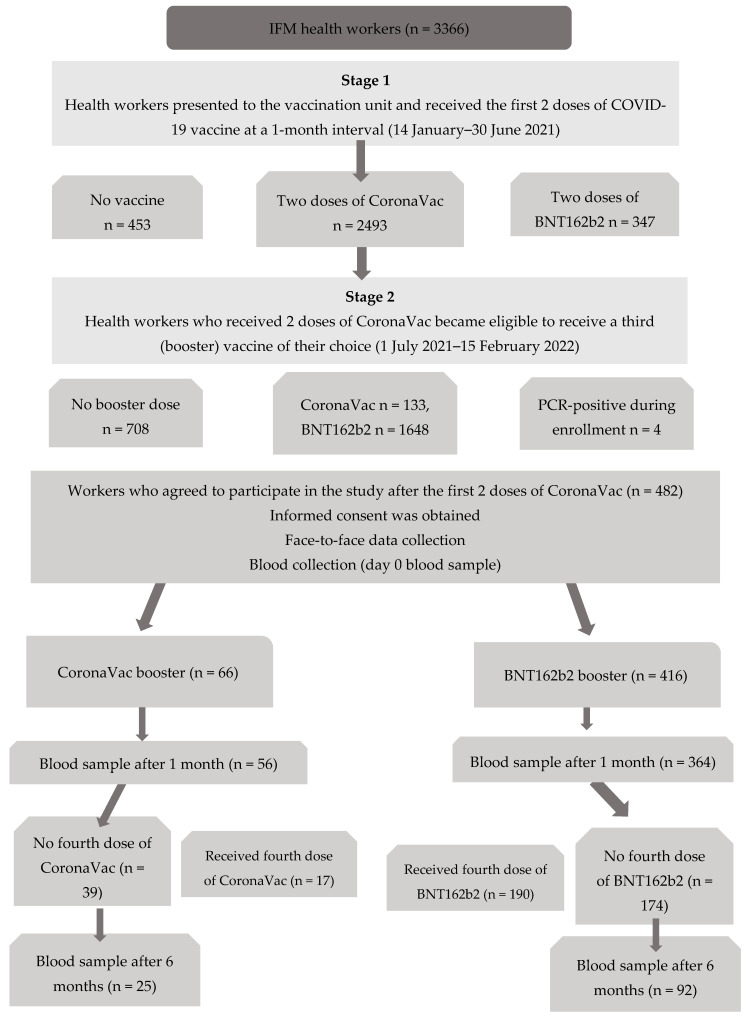
Flowchart of the participants in the cohort study.

This study represents the second phase of the vaccine cohort research and was completed between 1 July 2021 and 15 February 2022. A flowchart for the entire research process is presented in Figure 1. Our hospital employs 3366 staff. The research cohort consists of 2493 Istanbul Faculty of Medicine (IFM) Hospital employees who were followed-up in the Vaccination and Employee Health Outpatient Clinic since vaccination began on 14 January 2021 and continued to be followed-up after vaccination. A total of 712 employees who did not receive a booster dose or were PCR-positive for SARS-CoV-2 during the enrollment period were not included in this study. The resulting study group consisted of 1781 health workers. This study included health workers who presented for a booster vaccination at least 90 days after their second dose of CoronaVac, selected as well as received a booster vaccine dose, and volunteered to participate in the study. Study enrollment was conducted over a 30-day period. Participants who provided informed consent were followed for 6 months. Those who received additional booster vaccines during the follow-up were excluded from the study. Blood samples were collected from the participants at 1 and 6 months after they received the booster dose. Those who did not provide samples at these time points were also excluded (Figure 1).

### 2.2. Data Collection

All of the participants were informed about the study, and their informed consent was obtained. A questionnaire was administered in face-to-face interviews with the participants, and blood samples were obtained before the booster vaccination. The questionnaire included items regarding the participants’ sociodemographic characteristics (e.g., age, sex, occupation, and unit), comorbidities (e.g., diabetes mellitus, hypertension, congestive heart disease, chronic obstructive pulmonary disease, and chronic kidney disease), and history of COVID-19 infection within the 4 weeks prior to the booster vaccination. The influenza and pneumonia vaccinations received by the participants in the year before the study date were determined from their records. The participants were later contacted by phone to obtain blood samples at 1 and 6 months after the booster vaccination. In each call they were asked whether they had contracted a COVID-19 infection since receiving the booster vaccine. All data related to COVID-19 infection history were based on the participants’ self-reports.

### 2.3. Vaccines

#### 2.3.1. Inactivated SARS-CoV-2 Vaccine (CoronaVac)

CoronaVac^TM^ is an inactivated SARS-CoV-2 vaccine (Vero cell) produced by Sinovac Biotech Ltd. (Sinovac Life Sciences, Beijing, China). It was the first vaccine to be administered in our country by the Ministry of Health, starting on 14 January 2021. At the start of the vaccination program, the Ministry of Health recommended two doses at an interval of four weeks, and health workers were vaccinated accordingly. CoronaVac comes in single-use, 0.5 mL vials. After being shaken well, it was administered intramuscularly to the left shoulder, at a 90-degree angle to the skin, to individuals over the age of 18 years while seated. People with a history of allergies were observed for 30 min, whilst others were for 15 min for possible allergic reactions [12].

#### 2.3.2. BNT162b2 Vaccine

The BNT162b2 (Comirnaty^®^) vaccine is manufactured by BioNTech Manufacturing GmbH, Germany. This vaccine is a nucleoside-modified messenger RNA (mRNA) encapsulated in lipid nanoparticles (LNPs) and enables the delivery of RNA into host cells to allow the expression of the SARS-CoV-2 spike (S) antigen. One vial (0.45 mL) of this vaccine contains six doses. It must be diluted before use; before dilution, the vial contains a white to off-white frozen solution that may include white to off-white opaque amorphous particles. One dose for individuals aged 12 years or older contains 30 μg of COVID-19 mRNA vaccine. In this study, it was administered intramuscularly to the left shoulder at a 90-degree angle to the skin while the recipient was seated. People with a history of allergies were observed for 30 min, and others were for 15 min for possible allergic reactions [13,14,15].

### 2.4. Evaluation of Immune Response

Blood samples of at least 5 mL were collected into gel clot activator (yellow top) tubes and stored upright at room temperature for 30 min to allow clotting. The samples were then centrifuged at 2000× *g* for 10 min and the serum was transferred into numbered Eppendorf tubes. Serum samples were stored at −20 °C until analysis.

IgG antibodies against the SARS-CoV-2 spike receptor-binding domain (anti-S-RBD) in the serum samples were determined by the chemiluminescent microparticle immunoassay (CMIA) method using the Atellica IM SARS-CoV-2 IgG (sCOVG) assay (Siemens Healthcare, Erlangen, Germany), which has 95% sensitivity and 99.9% specificity [16]. The assay provides semi-quantitative measurements in index (U/mL) values that are interpreted as reactive or non-reactive. Samples with an index < 1.00 are classified as non-reactive, and these patients are considered negative for SARS-CoV-2 IgG antibodies, whereas samples with an index ≥ 1.00 U/mL are classified as reactive and should be considered positive for SARS-CoV-2 IgG antibodies. In addition, the manufacturer states that the cut-off value of an index of 1.00 corresponds to a reference standard concentration of 21.80 binding antibody units (BAU)/mL. Therefore, index values were multiplied by 21.80 to convert the results into BAU/mL units. Atellica IM sCOVG test results < 21.8 BAU/mL were interpreted as negative, results of 21.8 BAU/mL as borderline, and results > 21.8 BAU/mL as positive.

### 2.5. Statistical Analysis

Continuous variables were tested for a normal distribution via using Kolmogorov–Smirnov and Shapiro–Wilk tests. Descriptive statistics were given as median and minimum–maximum values for continuous data because they did not show a normal distribution. Frequency and percentage were given for discrete data. Statistical comparisons of categorical data were made using chi-square tests. For numerical data, comparisons of more than two independent groups were made using the Kruskal–Wallis test followed by post hoc pairwise Mann–Whitney U tests with a Bonferroni correction, while comparisons of more than two dependent groups were made using Friedman’s test followed by a post hoc Wilcoxon test for pairwise comparisons. *p*-values lower than 0.05 within a 95% confidence interval were considered statistically significant. IBM SPSS Statistics version 21.0 (IBM Corp., Armonk, NY, USA) was used for the statistical analyses.

Ethics committee approval for the study was obtained from the Clinical Research Ethics Committee of the IFM (dated 8 December 2019, number 1287). After approval by the Ethics Committee, permission to conduct the study was obtained from the Chief Physician of Istanbul University IFM Hospital (dated 9 December 2019, number 253608).

## 3. Results

Of the 117 participants, 81 (69.2%) were women, 61 (52.18%) were aged 40 and over, 54 (46.2%) were working in internal medicine divisions, and 69 (59.0%) were other health workers. There was no difference between the participants who chose CoronaVac and BNT162b2 as their third vaccine dose in terms of chronic diseases, influenza and/or pneumococcal vaccination status, history of COVID-19 infection, or IgG titer before booster vaccination (*p* > 0.05); however, a higher proportion of participants in the BNT162b2 booster group were women (*p* = 0.035) and in the 20–39 age group (*p* = 0.007) (Table 1).

Twenty-seven (23.1%) of the participants had a chronic disease. The most common chronic disease was hypertension (n = 9), followed by diabetes mellitus (n = 7), chronic lung disease (n = 6), asthma (n = 4), hypothyroidism (n = 3), Hashimoto’s thyroiditis (n = 1), and chronic cardiovascular disease (n = 1).

Comparisons of the IgG levels of participants boosted with CoronaVac and BNT162b2 according to month and selected sociodemographic characteristics are presented in Table 2. 

Of the participants boosted with CoronaVac, 64.0% did not contract COVID-19 at any time during the follow-up period, 12.0% had COVID-19 before the booster vaccination, and 32.0% had COVID-19 after the booster vaccination. Among those boosted with BNT162b2, these rates were 58.7%, 14.1%, and 33.7%, respectively (Table 1). Pre-booster antibody levels were similar between the two vaccine groups regardless of COVID-19 history; however, participants who received the BNT162b2 vaccine had significantly higher antibody levels at 1 month. At 6 months, the BNT162b2 booster group still had significantly higher antibody levels overall (*p* = 0.015) and among those with a negative COVID-19 infection history (*p* = 0.015), while there was no difference in antibody levels between the two vaccine groups among participants with a positive COVID-19 infection history (*p* = 0.708) (Table 3).

**Figure 2:**
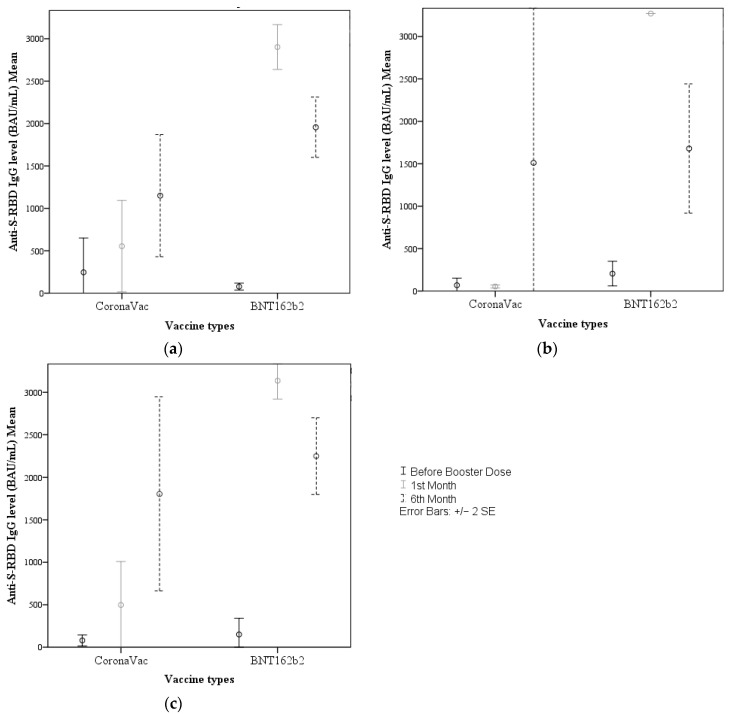
Comparison of total anti-spike IgG levels (BAU/mL) according to the history of COVID-19 infection, vaccine type, and follow-up month (**a**). Participants in the study cohort with no history of COVID-19 during the follow-up period (**b**). Participants in the study cohort who had COVID-19 before the booster vaccination (**c**). Participants in the study cohort who had COVID-19 after the booster vaccination.

Of the participants in the study cohort with no history of COVID-19 during the follow-up, those boosted with CoronaVac and BNT162b2 showed no difference in antibody levels in the pre-booster assessment (24.4 (10.9–3270) vs. 24.7 (10.9–707.6), *p* = 0.729), while those boosted with BNT162b2 had significantly higher IgG levels at 1 and 6 months after the booster (88.5 (0.0–3270.0) vs. 3270.0 (0.0–3270.0), *p* < 0.001 and 167.4 (11.6–3270.0) vs. 2255.2 (44.9–3270.0), *p* = 0.007, respectively) (Figure 2a).

Among the participants who reported contracting COVID-19 before the booster vaccination, there was also no statistically significant difference in IgG levels between the two vaccine groups pre-booster and at 6 months after the booster assessments (45.1 (10.9–149.9) vs. 94.2 (10.9–758.4), *p* = 0.458 and 1231.5 (29.9–3270.0) vs. 1618.2 (81.3–3270.0), *p* = 0.786, respectively), whereas those boosted with BNT162b2 had significantly higher IgG levels at 1 month after the booster (58.2 (36.6–65.8) vs. 3270.0 (3270.0–3270.0), *p* < 0.001) (Figure 2b).

The same pattern was observed among the participants who reported contracting COVID-19 after the booster vaccination, with no statistically significant difference between the vaccine groups pre-booster and at 6 months after the booster (49.7 (10.9–272.3) vs. 40.8 (10.9–2979.8), *p* = 0.68 and 2250.7 (28.1–3270.0) vs. 3270.0 (81.3–3270.0), *p* = 0.364), and a significant difference in favor of BNT162b2 at 1 month (144.6 (36.6–1995.8) vs. 3270.0 (0.0–3270.0), *p* < 0.001) (Figure 2c). Additionally, in those who received the CoronaVac or BNT162b2 vaccine, there was no difference between the antibody levels of “those with and without COVID infection” before the booster dose and “those with and without COVID infection” after the booster dose (*p* > 0.05).

## 4. Discussion

This study, which aimed to evaluate vaccine superiority by analyzing the antibody responses of health workers who were initially vaccinated with CoronaVac (two doses one month apart) and later received a booster dose of either CoronaVac or BNT162b2, presents the 6-month follow-up results of a prospective cohort research including 2493 IFM employees.

Ensuring widespread access to safe and effective vaccines is essential in a pandemic. The priority vaccination of health workers, who are in the highest-risk group, is an important step in terms of controlling the pandemic, supporting “herd immunity”, and ensuring the continuity of health services [17]. As CoronaVac was the only vaccine available at the time, it was used at the start of the vaccination program. When the BNT162b2 vaccine was later introduced, both options were presented for booster vaccinations [2]. Thus, health workers in our country were given the right to choose their booster vaccine after receiving two doses of CoronaVac.

In a study on health workers conducted by Çağlayan et al. [18], it was observed that, at 4 months, 79.8% of the participants showed a decline in antibody levels, with a mean decrease of 61.4% ± 20.0%. The gradual decrease in antibody levels observed with both the CoronaVac and BNT162b2 vaccines [19] reveals the importance of the booster dose. In another Turkish study conducted by Kara et al. [20], antibody concentrations were decreased but still detectable at 1 and 3 months after vaccination with two doses of the CoronaVac vaccine. Health workers in Turkey were allowed to choose which vaccine they wanted to receive as a booster dose. In this cohort study evaluating the effect of booster vaccine preference on antibody levels, it is important that the groups were similar in terms of independent variables, such as having chronic diseases and a history of COVID-19 infection, which are confounding factors that may affect antibody levels. In addition, the serum levels of anti-spike IgG were measured before the booster vaccination and were found to be similar between the two groups.

The protective efficacy of antibodies formed after vaccination or infection is determined by their neutralizing activity. Neutralizing antibodies bind to the pathogenic surface proteins that mediate adhesion to the host cell, thereby preventing conformational change and intracellular entry. Thus, the immune system provides protection without needing to summon immune cells. Antibodies without neutralizing activity, called binding antibodies, use other antibody functions, such as opsonization and complement system activation, to eliminate pathogens. The main neutralizing antibodies for SARS-CoV-2 are IgG antibodies against the virus’s S1 protein. As S1 binds to host cells’ ACE2 protein, it is key for the virus to enter human cells. Although other Ig classes or antibodies against other virus proteins, such as nucleocapsid proteins, may also have neutralizing effects, there are few sufficiently powered studies demonstrating the neutralization of SARS-CoV-2 virus in the absence of anti-S1 IgG antibodies [21]

Neutralization activity is determined by reference gold-standard tests that evaluate the presence of antibodies that inhibit the infection of cultured cells exposed to a live virus; however, neutralization tests must be conducted in biosafety level 3 laboratories and are time- as well as labor-intensive, making them difficult to standardize and apply on a large scale. Therefore, many studies have compared S1 IgG antibodies, which are largely responsible for neutralizing activity, with neutralization tests [22]. In a study comparing high-sensitivity and high-specificity automated CLIA tests to neutralization tests, all of the CLIA tests were able to identify circulating antibodies 12 days after vaccination, although the tests correlated over a wide range of dynamics. This study drew attention to the standardization of CLIA tests [23].

In another study comparing three different commercial anti-SARS-CoV-2 IgG detection platforms with the neutralization test, the Siemens kit used in our study showed the highest agreement (97.1%, 95% CI: 95.9–98.4%). The IgG values from each of these commercial kits were positively correlated with plaque reduction/neutralization antibody tests (R^2^ = 0.43/0.68 for Abbott, R^2^ = 0.57/0.85 for Euroimmun, and R^2^ = 0.39/0.63 for Siemens). Based on these results, the researchers recommended IgG serological tests as a practical approach in cases where neutralization tests are not available to evaluate immunity after infection and vaccination [24].

A study published in the Journal of Medical Virology evaluated the level of correlation between Euroimmun anti-SARS-CoV-2 QuantiVac ELISA (IgG) and a microneutralization test and revealed a strong positive correlation between anti-S1 IgG levels and neutralizing antibody titers (rs = 0.819, *p* < 0.001) [25]. In light of these studies published in reputable journals, we believe that, under circumstances in which neutralization tests are not accessible, determining the immune status of health workers after vaccination by detecting SARS-CoV-2 IgG antibodies via using a serological test with well-optimized sensitivity as well as specificity is important for the development of national strategic plans for protective measures during the pandemic.

When the CoronaVac and BNT162b2 groups were examined separately, we observed significant increases in IgG levels after the booster vaccination among all of the participants, regardless of whether or not they had a history of COVID-19 infection. This important finding demonstrates that both vaccines induce an antibody response; however, when IgG levels were compared between the two booster vaccines, there were no differences in pre-booster antibody levels, while post-booster antibody levels were significantly higher after the BNT162b2 vaccine at 1 and 6 months in all of the participants and at 6 months in participants with a negative history of COVID-19. Consistent with the literature, this finding demonstrates that the BNT162b2 vaccine has greater immunogenic potential than the CoronaVac vaccine. Studies have shown that BNT162b2 is one of the most protective of the COVID-19 vaccines developed, with over 90% efficacy against infection, severe infection, infection requiring hospitalization, and mortality after the second dose [19]. There are also previous studies conducted on health workers indicating that a BNT162b2 booster vaccination after initial vaccination with two doses of CoronaVac produced a much stronger immune response than a booster with CoronaVac [26,27].

Vaccines reduce the risk of contracting a COVID-19 infection by an average of 70–90%. These rates of protection mean that people who are fully vaccinated against COVID-19 can still become infected [28]. On the other hand, the results of clinical studies on the BNT162b2 vaccine showed that the vaccine was 95% effective in preventing symptomatic laboratory-confirmed COVID-19 in individuals with no evidence of a previous SARS-CoV-2 infection [29]. In a study by Alhinai et al. [30], findings from 41 countries with the highest rates of COVID-19 vaccination showed that there were more COVID-19 cases among people vaccinated with CoronaVac and other vaccines than among those vaccinated with BNT162b2 in the first 6 months of 2021. Although the BNT162b2 vaccine is known to be more protective than the CoronaVac vaccine, the results of our study indicated that more than half of participants in both groups had no COVID-19 infection during the follow-up, and similar proportions of participants reported contracting COVID-19 before and after boosting with both vaccines. This suggests that health workers with different ages, sexes, and comorbidities demonstrated similar responses to vaccines, and that each vaccine elicited specific antibody responses.

In addition to health workers, the WHO also identified people with comorbidities as a priority group for vaccination [31]. This is because COVID-19 was observed to disproportionately affect patients with chronic diseases and other comorbidities. In a systematic review and meta-analysis study, it was reported that patients with pre-existing chronic disease had a higher risk of hospitalization and death from COVID-19 [32]. Having a chronic disease was also reported to increase acceptance of COVID-19 vaccines [33]. Our finding that participants with chronic diseases preferred the BNT162b2 vaccine more in this follow-up study suggests that health workers are less affected by the infodemic because they have easier access to accurate information. In February 2020, WHO Director-General Dr. Tedros drew attention to the “infodemic” experienced during the COVID-19 pandemic during a speech, with the following statement: “We’re not just fighting a pandemic; we’re fighting an infodemic.” Information pollution at the global level has caused widespread confusion among societies [34].

The use of mRNA vaccines is recommended for patients with chronic disease due to their lower levels of responses to COVID-19 vaccines and the higher efficacy of mRNA vaccines such as BNT162b2 compared to other vaccines [35,36,37]. In another study conducted in Turkey, Bayram et al. [37] measured antibody titers in health workers vaccinated with CoronaVac and found that antibody positivity and median antibody titers were significantly lower in those with chronic diseases compared to those without. In the present study, we determined that the antibody levels of participants with chronic diseases only increased significantly after being boosted with BNT162b2 but not CoronaVac, whereas participants without chronic diseases showed increased antibody levels after booster doses of both vaccines; however, the low proportion of participants with chronic comorbidities who preferred the CoronaVac vaccine may have caused the lack of a significant difference. There has been a similar situation regarding influenza and/or pneumococcal vaccination in the last year. According to the adult vaccination policy in Turkey, pneumococcal and influenza vaccines are provided free of charge to people aged 65 years or older and/or with chronic heart/lung/metabolic diseases, in addition to those in other risk groups [38]. Therefore, as with chronic diseases, the low number of people with influenza/pneumococcal vaccinations among those who preferred CoronaVac may explain why no difference was observed.

The magnitude of antibody responses to certain immunological stimuli depends on many factors, including sex and age. Sex-based differences in antibody responses are vaccine-specific. While women have greater potential than men to form antibodies against the influenza vaccine, older men show stronger antibody responses to both pneumococcal and Td/Tdap vaccines compared to women [39]. Although studies on the effects of sex on antibody responses are limited, Takahashi et al. [40] reported that women produce stronger cellular and humoral responses than men, and suggested that they may therefore show stronger immune responses after exposure to SARS-CoV-2. On the other hand, immune responses to vaccination may differ between young adults and older adults, especially those over the age of 80, because of immune aging [41]. Some studies also indicated that a young age and the female sex were associated with higher antibody titers after vaccination compared to other age groups and the male sex [42,43]. A study by Lustig et al. [44] showed that vaccines also induced high IgG antibody concentrations in the older population, but that these antibodies may be less neutralizing or take longer to become neutralizing. In the present study, age group and sex had no effect on the IgG response produced by either vaccine at 1 and 6 months after the booster dose. The fact that we observed no sex- or age-related differences in the present study may be attributable to the fact that 70 per cent of the study group were women and that the participants over 65 years of age were not working due to retirement.

In this study, there was no statistically significant difference in the pre-booster IgG levels of participants who did not contract COVID-19 at any time during the follow-up and those who contracted COVID-19 before or after the booster vaccination, whereas participants who did not contract COVID-19 at any time during the follow-up and received the BNT162b2 booster had higher IgG levels at 1 and 6 months than those who received a CoronaVac booster. This finding shows that the BNT162b2 vaccine has greater potential to induce IgG production; however, we observed no difference in COVID-19 infection rates between participants boosted with the BNT162b2 and CoronaVac vaccines. Natural infection-induced or vaccine-induced humoral immunity against SARS-CoV-2 has been shown to provide some degree of protection against reinfection and/or reduce the risk of clinically significant outcomes.

In addition to the role of systemic antibodies in humoral immunity, the mucosal immune response is also considered to be critical in reducing viral spread. Some recent studies have shown that IgA has superior antiviral properties against influenza and SARS-CoV-2 compared to IgG [45].

Sterlin et al. evaluated the humoral immune response in the serum, saliva, and bronchoalveolar lavage fluid of patients with severe COVID-19 disease. Their results suggested that the early neutralizing response to SARS-CoV-2 was dominated by IgA and that serum IgA was seven times more effective in viral neutralization than serum IgG. The researchers associated their findings with the expansion of IgA plasmablasts with mucosal homing properties [45].

A longitudinal study examining the serological responses of SARS-CoV-2 mRNA vaccine recipients showed that, in addition to IgG, antigen-specific IgA also reached levels effective in preventing infection and transmission; however, the authors reported that spike-specific serum IgA levels decreased significantly faster than spike-specific IgG (*p* < 0.002). For both IgG and IgA, the “recall” response (time to peak serum levels after the second/booster dose) was significantly shorter than the primary response (*p* < 0.03) [46].

The protective effect of most systemic vaccines against mucosal infection is based solely on the transudation of a few circulating IgA and IgG antibodies from the serum to the mucosa. In a study examining anti-S1 and -RBD antibody induction by the BNT162b2 and heterologous ChAdOx1-S/BNT162b2 vaccines, no detectable antibodies against SARS-CoV-2 were found in saliva, in contrast to other studies. Based on their data, the researchers concluded that SARS-CoV-2 mRNA and vector vaccines cannot induce a detectable protective mucosal immune response. One explanation for the seemingly inconsistent results may be that the IgA antibodies detected in serum were likely transuded plasma anti-S1 antibodies [47]. The above being the case, the measurement of systemic IgG levels is still valid in the evaluation of the humoral response after vaccination.

Seropositive survivors are estimated to have 89% protection against reinfection [11]. Vaccine efficacy is reported to be between 70% and 90% [28]. This emerging evidence suggests that the BNT162b2 vaccine has high potential to induce IgG production and that both vaccines may be effective in preventing asymptomatic infections that may lead to transmission. Our results also support previous reports that vaccination induces a much stronger antibody response in patients with a history of COVID-19 infection and especially those who receive the BNT162b2 vaccine [48,49].

## 5. Conclusions

The results of this prospective cohort study showed the following:

- The BNT162b2 vaccine results in higher IgG levels than the CoronaVac vaccine among individuals with a positive history of COVID-19;
- The BNT162b2 vaccine was preferred by health workers with chronic diseases;
- Health workers are less affected by the infodemic because they have easier access to accurate information;
- In participants without chronic diseases, antibody levels increased significantly after both booster vaccines, but among participants with chronic diseases only those boosted with BNT162b2 showed a significant increase in antibody levels;
- Blood samples obtained before and after (1 and 6 months) booster vaccinations demonstrated no difference in the potential to induce an IgG response according to age group or gender;
- In terms of IgG responses to the two booster vaccines in participants without a history of COVID-19 and the entire participant group, no differences were observed within these groups before receiving the booster, whereas all participants who received a BNT162b2 booster had significantly higher IgG levels at 1 and 6 months. Among participants with a history of COVID-19 infection, there was no difference in pre-booster and 6-month IgG levels, while a significant difference was found in favor of BNT162b2 at 1 month.

Considering these results, health workers in the high-risk group and those with chronic diseases should prefer the BNT162b2 vaccine because of its higher potential to induce antibody production.

In conclusion, considering the gradual decline in immune response and the development of SARS-CoV-2 variants, our results suggest that even a single booster dose of the BNT162b2 vaccine after initial vaccination with CoronaVac provides a protective advantage from COVID-19 after 6 months, especially for risk groups such as health workers and those with chronic diseases.

This study has several limitations. Firstly, as the sample consisted of health workers who volunteered to participate, the study is susceptible to selection bias. Secondly, although the study was conducted in one of the largest tertiary hospitals in Turkey, the inclusion of only one center may impact the representativeness of the study. Thirdly, the participants were predominantly younger adults, and 69.0% were women. In addition, COVID-19 infection status data were based on self-reports and did not include information regarding the severity of infection. Therefore, our findings, pertaining to the effect of infection on post-vaccination IgG antibody titers, should be interpreted carefully. Finally, having only 117 participants complete the planned follow-up in its entirety also may have affected the results. The proportion of patients boosted with CoronaVac who had chronic diseases and pneumococcal/influenza vaccinations was very low. This should be taken into account when evaluating the results of the analysis of this subject.

## Figures and Tables

**Table 1:** Sociodemographic characteristics of the participants according to booster vaccine type.

**Sociodemographic Characteristics**	CoronaVac (n = 25)		BNT162b2 (n = 92)		*p*
	Number	% *	Number	% *	
Gender					0.035 ^a^
Male	12	48.0%	24	26.1%	
Female	13	52.0%	68	73.9%	
Age Group					0.007 ^a^
20–39	6	24.0%	50	54.3%	
≥40	19	76.0%	42	45.7%	
Department					-
Basic medical sciences	7	28.0%	9	9.8%	
Internal medicine divisions	7	28.0%	47	51.1%	
Surgical divisions	6	24.0%	27	29.3%	
Other	5	20.0%	9	9.8%	
Occupation					-
Physician	2	8.0%	14	15.2%	
Nurse	4	16.0%	22	23.9%	
Other health worker	17	68.0%	52	56.5%	
Office worker	2	8.0%	4	4.4%	
Chronic Disease					0.138 ^a^
No	22	88.0%	68	73.9%	
Yes	3	12.0%	24	26.1%	
Vaccination Status					
Influenza/Pneumococcal Vaccine					0.433 ^a^
No	18	72.0%	73	79.3%	
Yes	7	28.0%	19	20.7%	
History of COVID-19 Infection					
None	16	64.0%	54	58.7%	0.631 ^a^
Before booster	3	12.0%	13	14.1%	0.783 ^a^
After booster	8	32.0%	31	33.7%	0.873 ^a^
IgG titer before booster (BAU/mL), median (min–max)	31.2 (10.9–3270.0)		28.8 (10.9–2979.8)		0.88 ^b^

* Column percentage, ^a^ chi-square test, and ^b^ Mann–Whitney U test.

**Table 2:** Total anti-spike IgG levels (BAU/mL) according to vaccine type, month, and participant characteristics.

**Sociodemographic Characteristics**	CoronaVac (n = 25)				BNT162b2 (n = 92)			
	Before Booster (BAU/mL)	One Month after Booster (BAU/mL)	Six Months after Booster (BAU/mL)	*p* ^a^	Before Booster (BAU/mL)	One Month after Booster (BAU/mL)	Six Months after Booster (BAU/mL)	*p* ^a^
**Gender**								
Male	34.4 (10.9–3270.1)	88.5 (0–3270.0)	438.2 (11.6–3270.0)	**0.121**	29.9 (10.9–758.4)	3270.0 (0.0–3270.0)	2769.1 (44.9–3270.0)	**<0.001**
Female	17.7 (10.9–150.0)	115.3 (36.6–1223.4)	1231.5 (15.0–3270.0)	**0.009**	28.8 (10.9–32979.8)	3270.0 (0.0–3270.0)	2392.0 (81.3–3270.0)	**<0.001**
*p* ^b^	0.722	0.828	0.911		0.538	0.693	0.768	
**Age Groups**								
20–39 years	43.4 (10.9–3270.0)	124.7 (0.0–3270.0)	83.7 (28.1–3270.0)	0.738	27.4 (10.9–758.4)	3270.0 (0.0–3270.0)	2159.0 (44.9–3270.0)	**<0.001**
≥40	17.7 (10.9–272.3)	104.0 (36.6– 3270.0)	1231.5 (11.6–3270.0)	**<0.001**	31.5 (10.9–2979.8)	3270.0 (0.0–3270.0)	3270 (116.6–3270.0)	**<0.001**
*p* ^b^	0.262	0.874	0.648		0.55	0.346	0.207	
**Chronic Disease**								
No	24.4 (10.9–3270.0)	93.3 (0.0–3270.0)	518.2 (11.6–3270.0)	**0.001**	27.9 (10.9–3270.0)	3270.0 (0.0–3270.0)	2246.7 (44.9–3270.0)	**<0.001**
Yes	42.7 (10.9–278.6)	104.0 (73.0–191.2)	3270.0 (80.0–3270.0)	0.717	36.8 (10.9–612.6)	3270.0 (0.0–3270.0)	3270.0 (140.6–3270.0)	**<0.001**
*p* ^b^	0.643	0.738	0.266		0.179	0.645	0.262	
**Influenza/** **Pneumococcal** **Vaccination Status**								
No	17.5 (10.9–272.3)	87.6 (0.0–3270.0)	167.4 (11.6–3270.0)	**0.001**	29.9 (10.9–2979.8)	3270.0 (0.0–3270.0)	3067.0 (44.9–3270.0)	**<0.001**
Yes	53.2 (10.9–3270.0)	115.3 (36.6–3270.0)	2669.4 (22.7–3270.0)	0.772	23.1 (10.9–466.5)	3270.0 (0.0–3270.0)	1427.2 (140.6–3270.0)	**<0.001**
*p* ^b^	0.12	0.785	0.321		0.35	0.437	0.166	

^a^ Freidman test, ^b^ Mann–Whitney U test.

**Table 3:** Total anti-spike IgG levels (BAU/mL) according to vaccine type, month, and history of COVID-19 infection.

History of COVID-19 Infection	Booster Vaccine	Before Booster Dose (BAU/mL)	One Month after Booster (BAU/mL)	Six Months after Booster (BAU/mL)	*p* ^a^
Negative history (n = 70)	CoronaVac (n = 16)	24.4 (10.9–3270.0)	88.5 (0.0–3270.0)	167.4 (11.6–3270.0)	**0.019**
	BNT162b2 (n = 54)	24.7 (10.9–707.6)	3270.0 (0.0–3270.0)	2255.2 (44.9–3270.0)	**<0.001**
	*p* ^b^	0.729	**<0.001**	**0.007**	
Positive history (n = 47)	CoronaVac (n = 9)	45.1 (10.9–272.3)	115.3 (0.0–1995.8)	3270 (11.6–3270.0)	**0.045**
	BNT162b2 (n = 38)	41.4 (10.9–2979.8)	3270.0 (0.0–3270.0)	2874.8 (81.3–3270.0)	**<0.001**
	*p* ^b^	0.683	**<0.001**	0.708	
All participants (n = 117)	CoronaVac (n = 25)	31.2 (10.9–3270.0)	104.0 (0.0–3270.0)	788.9 (11.6–3270)	**0.001**
	BNT162b2 (n = 92)	28.8 (10.9–2979.8)	3270.0 (0.0–3270.0)	2469.4 (44.9–3270.0)	**<0.001**
	*p* ^b^	0.88	**<0.001**	**0.015**	

^a^ Freidman test, ^b^ Mann–Whitney U test.

## Data Availability

The data that support the findings of this study are available from the corresponding author upon reasonable request.
